# The earliest fossil cetacean with *Osedax* borings: narrowing the spatiotemporal gap between Cretaceous marine reptiles and late Cenozoic whales

**DOI:** 10.1098/rsos.250446

**Published:** 2025-06-11

**Authors:** Sarah Jamison-Todd, Philip D. Mannion, Paul Upchurch

**Affiliations:** ^1^ Department of Earth Sciences, University College London, London, UK; ^2^ Department of Life Sciences, Natural History Museum, London, UK

**Keywords:** *Osedax*, fossil cetaceans, bioerosion, Cenozoic, marine ecosystems

## Abstract

Borings of the extant bone-eating worm *Osedax* have previously been found in Cenozoic cetaceans and Cretaceous marine reptiles. The stratigraphically youngest Cretaceous example is from the Maastrichtian, and, until now, the oldest Cenozoic example was from the Oligocene. This leaves a substantial temporal and taxonomic gap between examples from both *Osedax-*hosting tetrapod groups. Here, we report nine fossil cetacean specimens with *Osspecus* (*Osedax* bioerosion), identified via CT scans. These include a late Eocene occurrence of the basilosaurid *Zyghorhiza kochii* from the eastern USA, which represents the earliest known Cenozoic occurrence of *Osedax* borings, narrowing the temporal gap between occurrences of *Osspecus* in Cretaceous marine reptiles and Cenozoic whales. These specimens also include the first *Osspecus-*bearing fossil cetaceans from the northwestern Atlantic, expanding the Cenozoic biogeography of *Osedax*. Six ichnospecies of *Osspecus* are found in these cetacean fossils, including one newly described ichnospecies. The high morphological diversity of *Osspecus* in these Cenozoic specimens is broadly consistent with that of the Late Cretaceous, with several ichnospecies now known from both time intervals. Surviving lineages of other large marine vertebrates, such as turtles, crocodyliforms and fish, likely acted as suitable resources for *Osedax* across the Cretaceous–Paleogene boundary, bridging both the temporal and taxonomic gap.

## 1. Introduction

*Osedax* [1] is a genus of bone-eating siboglinid annelid worm commonly found today on whale carcasses on the ocean floor [2], and is represented by 34 named living species [3,4] (World Register of Marine Species (WoRMS); https://marinespecies.org). There are also many additional undescribed species that are as yet unpublished, several of which have available genetic information but no species descriptions (GenBank: ncbi.nlm.nih.gov). This genus has an extensive geographic distribution and is tolerant of a wide range of depths and oxygenated marine environments [2] (Global Biodiversity Information Facility (GBIF); https://www.gbif.org). *Osedax* possesses highly derived adaptations for breaking down and gaining nutrition from bone material; in the absence of a digestive system, the worm uses bacterial mutualistic endo-symbionts acquired from the environment to obtain nutrition from lipids and/or collagen within the bone matrix [1,5,6]. This process creates borings that have a single aperture and a branching chamber ranging from approximately 3 to 10 mm in diameter that sits beneath the surface of the bone [7,8]. Modern whalebone has provided the basis for the original descriptions of these borings, but fossil examples have also been identified, classed within the ichnogenus *Osspecus* [9]. Different species of *Osedax* generally create different boring geometries, defined by variations in chamber morphology that can only be conclusively determined via CT-scanning [8]. Where geometries are distinct from one another, they may be used as a baseline proxy for species diversity both today and in the fossil record [8]. This allows descriptions of fossil *Osedax* borings to be used to trace the changes in species diversity and the distribution of the boring behaviours of *Osedax* through deep time.

Since the discovery of *Osspecus* in a range of Cretaceous marine reptile remains from Europe and North America that span the Albian to Maastrichtian [10–13], it has been clear that *Osedax* or its osteophagous ancestors were able to consume other large marine vertebrates prior to the origin and radiation of large marine cetaceans in the Eocene [14]. Eight ichnospecies have been named within the ichnogenus *Osspecus* [9,12,13] and many of these ichnospecies have geometries that are consistent through geological time, with some examples from Cretaceous marine reptiles bearing a strong resemblance to examples from fossil and/or present-day whales [12,13].

Convincing examples of *Osspecus* from the cetacean fossil record, identified by CT-scanning, have been discovered in late Miocene (G. Serafini, personal communication) and Pliocene remains from Italy [9], and early Oligocene specimens from Washington State, USA [15,16]. No instances from fossil whale bones older than the early Oligocene have previously been reported. The gap in the fossil record of *Osspecus* was, until now, between the Maastrichtian and Oligocene, a period of approximately 30 million years.

Here we present several *Osspecus-*bearing fossil cetacean occurrences from the east coast of the USA and from The Netherlands. Most notably, this includes material from the late Eocene of the USA, narrowing the temporal gap in the fossil record of *Osedax* borings and providing the earliest occurrence of *Osspecus* from the cetacean fossil record. Alongside the stratigraphically younger remains presented herein, these also represent the first described examples of *Osspecus* in cetaceans from the eastern seaboard of the USA. Additionally, Mio-Pliocene material from The Netherlands contains evidence for a newly recognized ichnospecies of *Osspecus*. Altogether, this new material helps to refine our understanding of both the biogeographic history and species diversity of *Osedax* in the spatial and temporal gap between previously known examples.

## 2. Material and methods

### 2.1. Institutional abbreviations

FMNH: Field Museum of Natural History, Chicago, IL, USA; MAB: Oertijdmuseum, Boxtel, The Netherlands; NHMUK: Natural History Museum, London, UK; USNM: United States National Museum of Natural History, Washington, DC, USA.

### 2.2. Materials and stratigraphic context

#### 2.2.1. FMNH PM 459

This partial skeleton of the basilosaurid *Zyghorhiza kochii* is the oldest cetacean specimen presented here, and comes from the Pachuta Marl Member of the Yazoo Formation in Alabama, USA [17]. Due to a hiatus in deposition that straddles the Eocene–Oligocene boundary, this specimen may be either Priabonian (uppermost Eocene) or from the Eocene–Oligocene boundary [17]. This stratigraphic horizon represents a shallow marine environment of the Gulf Coastal Plain on the eastern seaboard of North America [17]. The skeleton shows bioerosion at the surface in many of the elements. Pitting is particularly heavy on the ribs, which may have remained elevated above the sediment surface during the decomposition and collapse of the skeleton. Two ribs and a neural arch were scanned. The ribs and neural arch are confirmed to belong to the specimen FMNH PM 459, and are labelled and catalogued as such (W. Simpson, personal communication), though they are not mentioned in the original Gingerich [17] paper describing the specimen [17].

#### 2.2.2. MAB 15991, MAB 15992, MAB 15993, MAB 16027 and MAB 16028

These five specimens are all single elements from unidentified odontocete cetaceans with varying degrees of bioerosion. MAB 15991−3 are weathered teeth, MAB 16027 is a partial vertebra and MAB 16028 is a partial tooth. These five elements come from sand layers of the Tortonian (Upper Miocene) Breda Formation and the overlying Piacenzian (Lower Pliocene) Oosterhout Formation in The Netherlands [18,19]. The material was dredged from a lake bottom and the stratigraphic provenance is poorly constrained, but the bones are from an offshore marine environment [18,19], likely the basal lag of the Oosterhout Formation [18,19]. Concurrent weathering suggests that some of them, particularly the large, cylindrical teeth, may have experienced a high degree of transport. MAB 15991 is heavily pockmarked on all sides, with many chambers of varying sizes ringed by filamentous branches, giving the pockmarks a frayed appearance. MAB 15992 has more intermittently distributed chambers and some ‘pinhole’ type surface bioerosion. MAB 15993 also has collapsed chambers of varying sizes distributed across the surface. MAB 16027−8 have ‘pinhole’ style bioerosion next to hemispherical chambers, often coinciding in patches on all sides of the vertebra. Many other bones from this locality are also bioeroded.

#### 2.2.3. USNM PAL 768907, USNM PAL 768908, USNM PAL 768909, USNM PAL 768910 and USNM PAL 768911

USNM PAL 768907−8 are teeth referred to *Physeterula* sp., USNM PAL 768909−10 are also teeth, cf. *Physeterula* sp., and USNM PAL 768911 is a small indeterminate odontocete radius. These isolated elements are recorded as sourced from the Mio-Pliocene Chesapeake Group in North Carolina, USA, with USNM PAL 76807 and USNM PAL 76808 recorded as coming from the middle Miocene Pungo River Formation, USNM PAL 76808 and USNM PAL 76809 recorded as from the Pliocene Yorktown Formation and USNM PAL 768911 having no additional provenance information. The fossils were retrieved from spoil piles, so the exact stratigraphic context is imprecise [20], and these fossils can therefore most accurately be described as Mio-Pliocene specimens from the Chesapeake Group. The bones were all found in the Lee Creek Mine locality, which has been a prolific site for odontocete cetaceans [20]. As with the Dutch material, they are from a shallow nearshore setting and some have undergone extensive re-working [20]. USNM PAL 68907−10 are bioeroded to varying degrees, USNM PAL 768908 and USNM PAL 768910, in particular, with a higher degree of bioerosion and more distinct outlines of radial ‘firework’-like chambers. USNM PAL 768911 has small pits and ‘pinholes’ scattered across the surface of the bone.

### 2.3. Data collection

**Figure 1.**
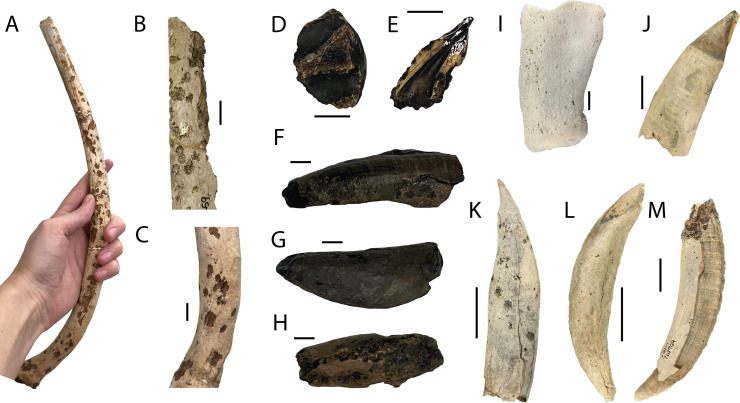
Bioeroeded cetacean bones in this study. (A) A rib from the late Eocene partial basilosaurid skeleton FMNH PM 459 showing heavy surface pitting similar to that found on many of the ribs. (B,C) Close-ups of bioerosion on the ribs of FMNH 459. Scale bars are 10 mm. (D–H) Cetacean elements from the Mio-Pliocene of The Netherlands with varying degrees of bioerosion visible at the surface. Scale bars are 10 mm. (D) MAB 16027. (E) MAB 16028. (F) MAB 15991. (G) MAB 15992. (H) MAB 15993. (I–M) Cetacean elements from the Mio-Pliocene of North Carolina with varying degrees of bioerosion at the surface. Note the ‘firework’ pattern on the tooth roots made by some of the *Entobia* borings. Scale bars are 20 mm. (I) USNM PAL 760911. (J) USNM PAL 760910. (K) USNM PAL 760907. (L) USNM PAL 760908. (M) USNM PAL 760909.

The material was first examined at the surface to determine if bioerosion was present. A selection of bones with surface bioerosion that resembled potential *Osspecus* were selected for CT-scanning (table 1). Collapsed hemispherical chambers that occurred in clusters and individually, with consistent shape and size, and round millimetre-scale apertures resembling pinholes were used as visual indicators of potential *Osspecus* (figure 1). Specimens from MAB, USNM and NHMUK were scanned at NHMUK with the Nikon Metrology HMX ST 225 micro-CT scanner, and those from FMNH were scanned with the University of Chicago PaleoCT (RRID:SCR_024763). All scans of the relevant specimens, including scan parameters, are available on MorphoSource: https://www.morphosource.org/projects/000669907?locale=en. For material from both the North Carolina and Netherlands Mio-Pliocene localities, a considerable amount of bioerosion is also present on the other collection material from these sites. For the North Carolina material, approximately 150 additional examples of probable invertebrate bioerosion were observed in collections, but only a select few bones were loaned for scanning on the basis that they were the most likely to show representative forms of bioerosion. In The Netherlands material, about 10 additional bones have similar bioerosion but were not scanned. These additional instances are proportional to the size of the collections from these localities and represent approximately 10% of the available material from these sites.

**Table 1.** Sampled cetacean specimens with geographic and stratigraphic provenance, as well as associated forms of bioerosion.

specimen number	cetacean element	stratigraphic unit	approximate age	bioerosion present
FMNH PM 459	*Zygorhiza kochii* partial skeleton	Yazoo Fm., Alabama	upper Eocene (Priabonian)	*O. tuscia, O. frumentum, O. panatlanticum, O. eunicefooteae*
MAB 15991	cetacean tooth	Breda/Oosterhout Fms., The Netherlands	Mio-Pliocene	*O. panatlanticum,* *O. pollardium,* *Entobia,* indet. tubes
MAB 15992	cetacean tooth	Breda/Oosterhout Fms., The Netherlands	Mio-Pliocene	*Entobia*, indet. tubes
MAB 15993	cetacean tooth	Breda/Oosterhout Fms., The Netherlands	Mio-Pliocene	*O. morsus,* *O. pollardium*
MAB 16027	partial cetacean vertebra	Breda/Oosterhout Fms., The Netherlands	Mio-Pliocene	*O. tuscia,* *O. morsus,* *O. eunicefooteae*
MAB 16028	partial cetacean tooth	Breda/Oosterhout Fms., The Netherlands	Mio-Pliocene	*O. morsus,* indet. tubes
USNM PAL 768907	*Physeterula* tooth	Pungo River Fm., NC	middle Miocene	*O. eunicefooteae,* *Entobia*
USNM PAL 768908	*Physeterula* tooth	Pungo River Fm., NC	middle Miocene	*O. eunicefooteae,* *Entobia*
USNM PAL 768909	odontocete tooth cf *Physterula* sp.	Yorktown Fm., NC	Pliocene	*O. tuscia,* *Entobia*
USNM PAL 768910	odontocete tooth cf *Physterula* sp.	Yorktown Fm., NC	Pliocene	*Entobia*
USNM PAL 768911	small odontocete radius	Chesapeake Grp	Mio-Pliocene	*O. tuscia*

### 2.4. Ichnotaxonomic identification

In the bones that were determined to have examples of invertebrate bioerosion (figure 1, table 1), the borings were classified based on their morphology. Without the distinct features of a single aperture and a branching chamber, identification of *Osspecus* is not definitive. Other marine bioeroders, such as clionaid sponges or small bivalves, can create borings in bone that are superficially similar to *Osedax* borings [9,21]. As such, CT-scanning is crucial for identification of *Osedax* borings, so that the complete three-dimensional and cross-sectional morphology of the boring can be examined, enabling differentiation between *Osspecus* and other bioerosive traces.

For examples of *Osspecus* in the cetacean material presented here, the morphology of the borings was compared to both present-day and fossil occurrences and, where possible, assigned to previously described ichnospecies after Jamison-Todd *et al*. [12,13] with one new ichnospecies described herein, using morphological features comprising: diameter and depth range of the borings measured in cross section; relative size and geometry of the aperture and aperture neck; relative depth of penetration into the bone; position of the centre of radial symmetry within the chamber; presence or absence of secondary radial symmetry in the branches; branch length and geometry; and overall chamber geometry.

**Figure 2.**
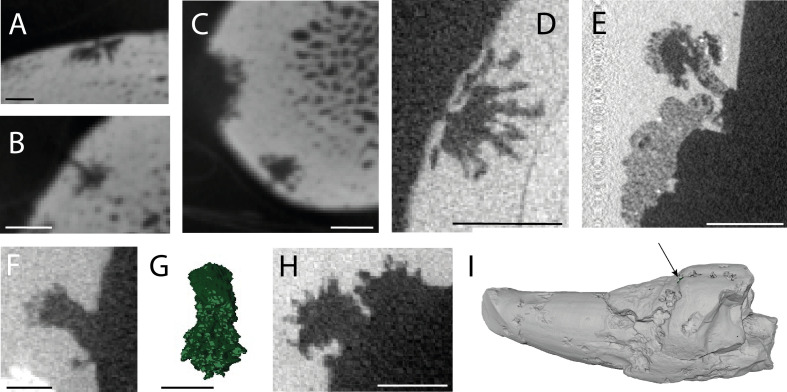
The five previously described ichnospecies and one new ichnospecies of *Osspecus* were found in the cetacean bones presented here. (A) *O. tuscia*, in FMNH PM 459. (B) *O. frumentum,* in FMNH PM 459. (C) *O. panatlanticum,* in FMNH PM 459. (D) *O. eunicefooteae,* in USNM PAL 768907. (E) *O. morsus,* MAB 16028. Scale bars in (A–E) are 2 mm. (F) Holotype boring of *Osspecus pollardium* in MAB 15993. (G) Three-dimensional reconstruction of holotype boring. (H) Additional representative examples of *O. pollardium* in MAB 15991. (I) Location in the bone of holotype boring. Scale bars in (F–I) are 1 mm.

**Figure 3.**
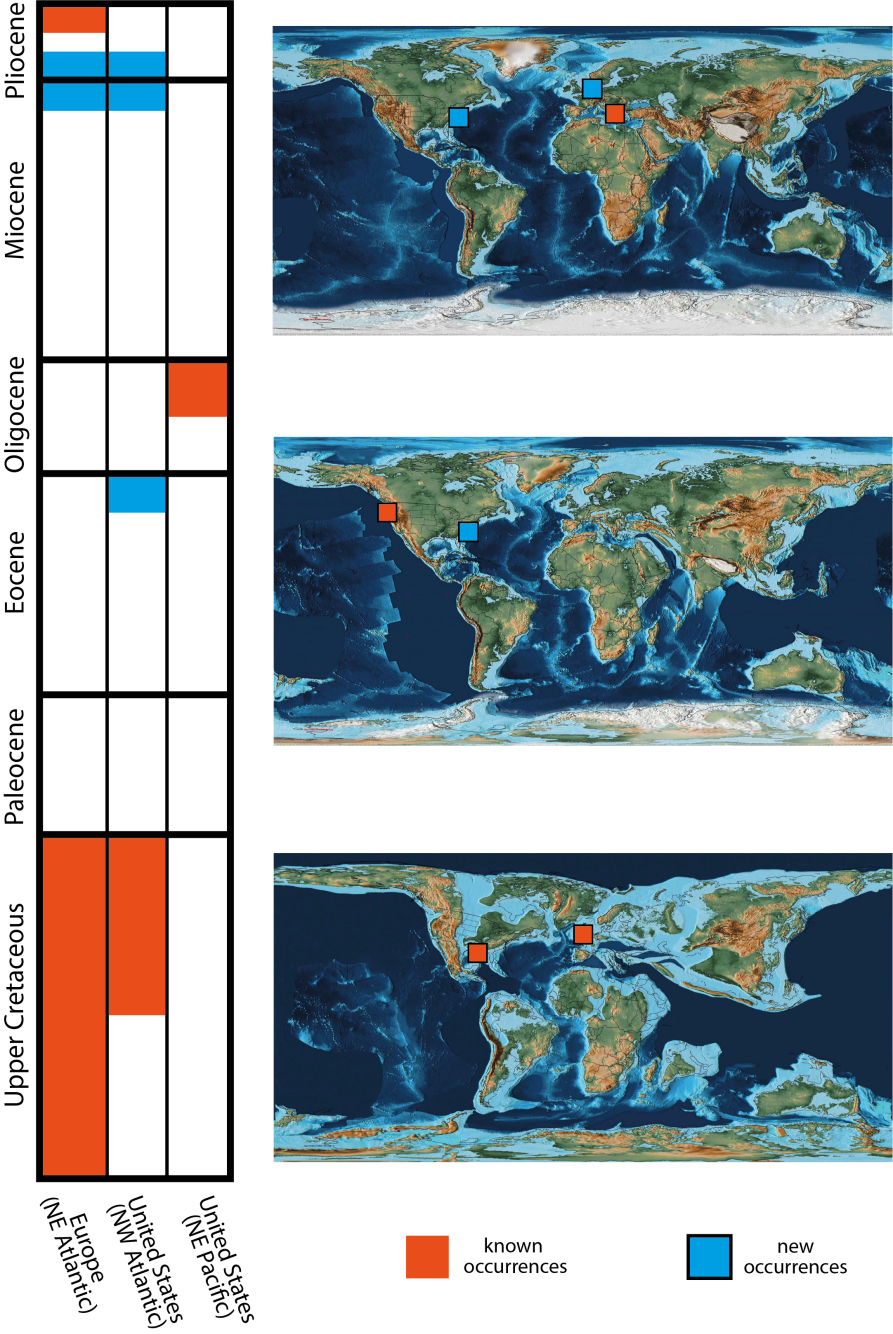
Occurrence data sectioned into temporal and geographic regions. The new occurrences (blue) of *Osspecus* (and inferred presence of *Osedax*) presented here help to fill in spatiotemporal gaps in the pre-existing data (orange). PALEOMAP PaleoAtlas maps [29], from top to bottom, represent the continental configurations of the Late Miocene, Late Eocene and Early Campanian. The top map contains occurrences from the Miocene and Pliocene, the middle map contains occurrences from the Eocene and Oligocene and the lower map contains occurrences from the Cretaceous. Colours represent temporal and geographic regions where occurrences have been found rather than individual number of occurrences.

Most borings made by sponges are referred to the ichnogenus *Entobia* [22], and have only twice previously been described in fossil bone material [23,24]. They consist of canals that may connect chambers to the surface and to each other, or may be more linear [22]. Generally, sponge borings are described in biogenic and non-biogenic carbonate substrates [25–27] (see Bromley & D’Alessandro, fig. 2 [26] and Beuck & Freiwald, fig. 3 [25] for examples). Branches of *Entobia* are also more filamentous than those of *Osspecus* and bifurcate repeatedly along their length, due to the morphology of the sponges themselves (see Chaves-Fonnegra & Zea, fig. 1 [28]). A comparison of these borings to those found in bone can help with diagnosis, despite differences in the density of bone and its composition of calcium phosphate rather than calcium carbonate. Sponges may make large chambers with filamentous branches [28], so that when weathered, identification of bioerosion is less certain. It should also be noted that substrate colonization by sponges can occur at any point in the depositional history of a fossil when it is exposed on the sea floor. That is, if a bone is re-exposed after fossilization, it may be colonized and bored secondarily by sponges.

For examples of bioerosion thought to belong to the ichnogenus *Entobia* in the material presented here, morphological diagnosis was made based on previous descriptions of sponge borings in carbonate and biogenic substrates and on the morphology of the sponges that create the borings, namely the main body and excavating filaments that bore into the substrate. For undiagnosed tube-like structures in the bones, measurements were also taken and the examples compared to those in the literature.

## 3. Results

### 3.1. Bioerosion occurrences

Eleven fossil cetacean specimens have invertebrate bioerosion, with clear examples of *Osspecus* found in nine of these specimens (table 1). FMNH PM 459 provides the earliest instance of *Osspecus* in a fossil cetacean. This late Eocene specimen and the four Mio-Pliocene specimens from the USNM provide the first fossil cetacean examples with *Osspecus* from the eastern seaboard of North America. Seven specimens also have bioerosion attributed to sponges and tentatively assigned to the ichnogenus *Entobia*, and additional tube-like structures form another type of unidentified bioerosion in three specimens (table 1).

Examples of *Osspecus* are classified based on the morphological descriptions of previously described ichnospecies, and all share the features that define this ichnogenus and are reflected in the morphology of the worm itself (figure 2) [1]. Six ichnospecies of *Osspecus* are identified in these bones, many of them co-occurring with one another (table 1). Five of these can be referred to previously described ichnospecies [9,12,13] (figure 2). One additional boring geometry is referred to a new ichnospecies (figure 2). The latter is described below, alongside spatiotemporal expansions of the other five ichnospecies found in the material presented herein (figure 3).

### 3.2. *Osspecus* occurrences

*Osspecus* igen. Higgs *et al*. [9]

*Osspecus pollardium* isp. nov

LSID: urn:lsid:zoobank.org:act:F6DCF229-671A-4CE1-B374-C5FBA2500342

Etymology: Named for its resemblance to a pollarded tree.

Holotype: Boring in MAB 15993.

**Figure 4.**
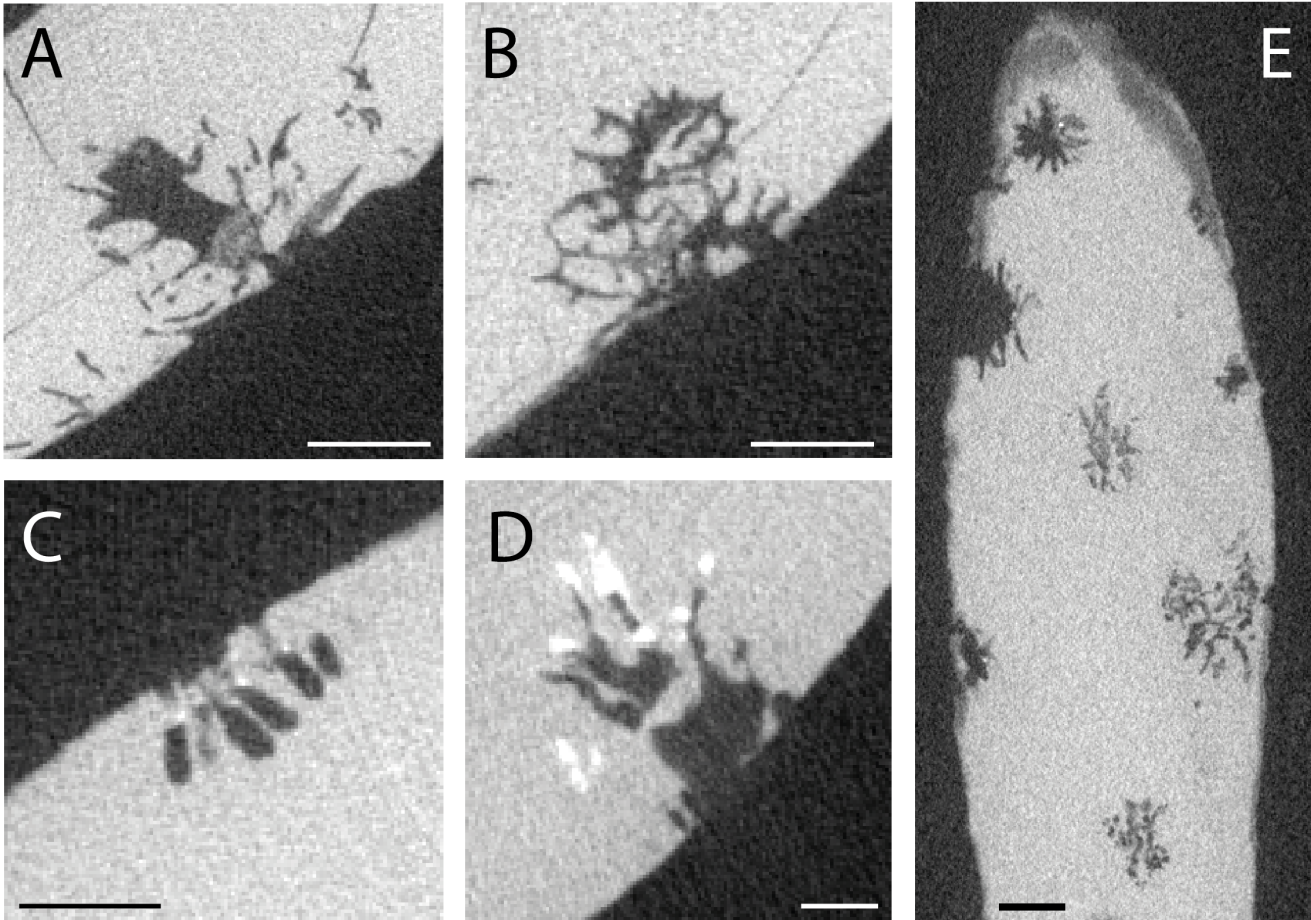
Other forms of bioerosion are found in the cetacean bones presented here. (A,B) Two cross-sectional slices of the same *Entobia* boring or boring cluster in USNM PAL 768910, showing the variation in morphology of the chambers and the right-angle branching of the filaments. (C) Unidentified linear structures in MAB 16028. (D) A cluster of unidentified tubes in MAB 15992. (E) Cross-section of USNM PAL 768907 showing the ‘firework’ pattern of the *Entobia* borings, which is also sometimes visible at the surface. Scale bars in (A,B,E) are 2 mm. Scale bars in (C,D) are 1 mm.

Figure 4, this study.

Diagnosis:

Borings with 1.1−2.2 mm total depth range, 0.7−2.8 mm chamber diameter range and 0.3−1.3 mm aperture width range. The aperture neck is wide relative to the chamber diameter, forming a column slightly narrower than the chamber diameter and leading into the wider main chamber. Thin, filamentous branches are distinct from this central chamber, radiating from its base into the surrounding bone.

Remarks:

These borings are commonly found in clusters that may represent multiple individuals exploiting the same weakened bone surface. There is a broad size range, but the geometry of the ichnospecies is highly distinctive. The borings are found only in two of the Mio-Pliocene teeth from The Netherlands, and therefore have a limited known temporal and geographic range thus far.

*Osspecus tuscia* [9]

This ichnospecies, the type of the ichnogenus *Osspecus*, was originally described in Pliocene whalebone from Italy [9]. It has since been found in three Late Cretaceous marine reptiles from the UK [12,13] and the occurrences newly presented here reduce the gap in the fossil record of the ichnospecies by providing late Eocene and Mio-Pliocene occurrences. The examples reported here also stem from both The Netherlands and the eastern USA, expanding the geographic range of the ichnospecies beyond Europe.

*Osspecus morsus* [12,13]

This ichnospecies is often, but not always, found in teeth: here, two out of three of the specimens containing *O. morsus* are teeth, whereas the third is a vertebra. All are from The Netherlands, and the ichnospecies is still constrained geographically to Europe, having previously been found only in the UK. The stratigraphic range, however, has been significantly expanded with these new examples, as this ichnospecies was previously described only in the Late Cretaceous, and here it is found in Mio-Pliocene deposits.

*Osspecus frumentum* [12,13]

Fossil specimens of this ichnospecies were previously limited to one instance in a pliosaur tooth from the Cenomanian of the UK [10,12,13]. Modern representation of this ichnospecies is based on traces in present-day whalebone from Monterey Canyon off the coast of California [8]. The new examples reported here are in a late Eocene basilosaurid from the eastern USA, representing the stratigraphically earliest instance of *Osspecus* in a cetacean, and therefore narrowing the gap both geographically and temporally between previously known occurrences of this ichnospecies.

*Osspecus panatlanticum* [12,13]

Modern examples of this ichnospecies are found in Monterey Canyon, off the coast of California [8], and also in the North Atlantic, off the coast of Sweden [7]. It is not clear whether these are made by the same species of *Osedax*, and the new examples presented here bear the strongest resemblance to the Californian borings. *Osspecus panatlanticum* was named for its presence in Late Cretaceous marine reptile bones from the eastern USA, as well as Belgium and the UK [12,13]. This ichnospecies is therefore widespread early in the fossil record of *Osspecus*. The Eocene whale with this ichnospecies provides an additional instance in the Atlantic Ocean basin since the Campanian.

*Osspecus eunicefooteae* [12,13]

The modern examples of this ichnospecies are made by *Osedax antarcticus* and were found off the coast of Smith Island, Antarctica [8,30]. Otherwise, only found in the Late Cretaceous of Europe until now, the spatiotemporal distribution of this ichnospecies is expanded by its presence in the late Eocene of the eastern USA, and it is also found in the Mio-Pliocene material from The Netherlands. These instances provide additional occurrences of *O. eunicefooteae* between the Cretaceous and the present day.

Emended diagnosis of *Osspecus eunicefooteae*

In addition to the previously identified and described features of this ichnospecies, such as long, undulating branches emanating radially from a central hemispherical chamber that sits near the bone surface [12,13], the new examples presented here sometimes have distinctly long, straight, streamer-like branches emanating from the central chamber down into the bone.

### 3.3. Additional bioerosion forms

Some of the borings present in the cetacean fossils presented here resemble borings made by clinoaid sponges in carbonate and biogenic substrates [25–28] and fit the ichnotaxonomic descriptions for *Entobia* [22,31] (figure 4). Some are represented by hemispherical chambers with filamentous branching, and others by more linear chambers with right-angle dendritic branching. These borings are often approximately twice the size of those identified as *Osspecus*, and are not consistent with the central chamber and radial symmetry of *Osspecus*. The bioerosion is instead consistent with the morphology of boring sponges, with the main body sending out excavating filaments into the substrate (Chaves-Fonnegra & Zea, fig. 1 [28]). *Entobia* occurs here in bones from the two Mio-Pliocene localities containing fossils that have been exposed on the seafloor for some time or have been re-worked (table 1).

Some of the bioerosion in the cetacean fossils could not be classified. This bioerosion often consists of tube-like structures either individually or in clusters, sometimes associated with hemispherical collapsed chambers that may be the result of weathering of bone made porous by bioerosion (figure 4). The borings resemble single or double tubes oriented approximately perpendicular to the bone surface, or clusters of thin tubes, generally less than half a millimetre in diameter. These bioerosive structures are limited in this study to The Netherlands Mio-Pliocene locality, and also to cetacean teeth (table 1). They are potentially exploratory and incomplete examples of *Osspecus* or *Entobia*, but this association is not clear. They may also be an entirely different form of bioerosion created by some unknown organism. These borings are superficially similar to those described in the teeth of a Middle Jurassic thalattosuchian crocodyliform (see Serafini *et al.*, fig. 10C,D [32]).

## 4. Discussion

### 4.1. Ecological preferences and depositional environments

The partial basilosaurid skeleton, FMNH PM 459, is unique not only in its stratigraphic age, as the earliest fossil whale with evidence of *Osspecus*, but also in terms of its geological setting among *Osspecus-*bearing fossil cetaceans. The stratigraphically younger occurrences reported here come from sandy re-worked deposits, as do the fossil whale examples from the literature [9,15,16]. FMNH PM 459, however, was preserved in a marl that represents a similar environment to the chalk seas and other shallow-marine carbonate environments from which most of the Cretaceous marine reptile instances of *Osspecus* originate [10]. These more productive nearshore environments may have been conducive to hosting multiple species of *Osedax* or its ancestors, with higher levels of oxygen and large vertebrate falls more readily available than in open ocean environments. It is possible that these environments were preferred by *Osedax* or its ancestors in the Cretaceous, but that the lineages leading to extant forms evolved a tolerance of a wider range of environments [2].

Preferential preservation of certain depositional environments should be taken into account, however, when interpreting the deep-time environmental preferences of *Osedax* and its predecessors. The preservational bias towards shallower environments in the rock record generally eliminates deep-water strata [33], and it is possible that there were deeper-water *Osedax* species than those whose borings have been found to date, which have not been preserved as *Osspecus* in the fossil record. Some Oligocene examples are particularly notable in that they are from bathyal depths [15,16]. Given that low- or no-oxygen environments often produce the best-preserved fossil vertebrate material, and therefore a high percentage of the museum collections material studied here, an additional preservational bias against the likelihood of finding fossil *Osspecus* is added. No examples have been found in the fossil record from anoxic or dysoxic environments, but this is likely a genuine signal, because living species of *Osedax* are not known to tolerate the absence of oxygen.

Nearly all the specimens presented here (except FMNH PM 459) are from re-worked deposits, where a high degree of transport has weathered the specimens and exposed them to other forms of bioerosion, such as *Entobia*, which is made by substrate-colonizing sponges exploiting hard bone clasts exposed on the seafloor. Given that a high percentage of the fossil material from The Netherlands and North Carolina is bioeroded, a long exposure time on the seafloor prior to burial and fossilization seems the most likely cause for the prevalence of *Osspecus*, whereas the secondary exhumation and re-working may have provided a window for sponge colonization at a later time.

### 4.2. Cenozoic diversity and biogeography

Through describing new instances of *Osspecus* bioerosion in fossil whalebone, we have been able to expand our knowledge of the deep-time narrative of *Osedax* diversity and biogeography (figure 3). This applies to individual ichnospecies, as well as to the genus as a whole. When compared to previous findings, these additional examples provide new data points both temporally and geographically. The ichnospecies that are found in FMNH PM 459, comprising *O. tuscia*, *O. frumentum*, *O. panatlanticum* and *O. eunicefooteae*, had only previously been found in Late Cretaceous marine reptiles [10–12] or in more recent (Pliocene to present day) whalebones [7–9]. This left a large temporal gap (of approx. 60 million years) in the data for these ichnospecies between the latest Cretaceous and the recent fossil record (i.e. the last 5 million years). The new evidence for Eocene examples of these ichnospecies adds data points to the earlier Cenozoic portion of their inferred ranges. *O. morsus*, previously known only in the Cretaceous [10,12,13], is now also found in the Mio-Pliocene, a significant time range extension for this ichnospecies. If certain taxonomic groups are responsible for the boring behaviours that persist through these ranges, then these subgroups or species of *Osedax* may also have persisted on these timescales.

On the generic scale, the approximately 40 million year temporal gap between the previously described latest Cretaceous [10] and earliest Cenozoic [15,16] examples of *Osspecus* now is supplemented by an expanded fossil record between the Maastrichtian and Oligocene, with the new instance in the late Eocene, exemplified by FMNH PM 459 (figure 3). Stem cetaceans originated in the early Eocene, but did not become fully aquatic until the middle Eocene [14,34], and *Osedax* or its ancestors presumably consumed the bones of other vertebrates between the end-Cretaceous extinction of most marine reptiles and the emergence of marine cetaceans. This is supported by the occurrence of living *Osedax* in a number of types of bone in marine environments today [3], and even on terrestrial vertebrate bones that settled in a marine environment [35]. *Osedax*-like borings have been found in Oligocene fish bone [16], modern shark teeth [36], and Cretaceous marine turtle bone [11]; representatives of all of these marine vertebrate groups survived across the Cretaceous–Paleogene (K-Pg) boundary, alongside lineages of crocodyliforms adapted for marine habitats [37–39], and it is possible that terrestrial vertebrate carcasses also contributed a food source for *Osedax* and/or its ancestors. Vertebrates do not necessarily need to be large-bodied to be colonized by *Osedax*, but the fossil record is more likely to preserve larger-bodied animals like marine reptiles and cetaceans. Larger-bodied animals are less likely to be completely dismantled by scavengers and current or wave energy, and take longer to decay, and are therefore more likely to survive sinking to the seafloor and being buried intact.

The new instance of *Osspecus* from the late Eocene presented here provides a slightly earlier example in the evolutionary history of cetaceans, from the previous earliest instance in the early Oligocene, with four individual ichnospecies found in FMNH PM 459. The number of ichnospecies in a single Eocene cetacean shows a relatively high diversity of likely *Osedax* species in very early aquatic whales. Rather than resulting from the origination of large marine cetaceans, we suggest that a high diversity of *Osedax* species had likely persisted from the Mesozoic and been sustained by other vertebrates in the interval between. It is possible that the Cretaceous borings were made by a separate lineage of bone-eating worms divergent from and not ancestral to the living genus *Osedax*, and the remaining gap between the latest Cretaceous occurrences and early Cenozoic occurrences might be real. However, there is a strong case for continuity rather than convergence of boring behaviours, because some of the same ichnospecies are found in the Cretaceous through to the Cenozoic and the present day [12,13]. Paleocene examples from other vertebrates would help to fill the remaining temporal gap and to confirm the continuity of *Osedax* from the Cretaceous to its extant form.

The biogeographic history of *Osedax* or its ancestors between the Campanian and Oligocene is also an important issue. In the Cretaceous, no examples have been found outside of the Atlantic Ocean basin, and the Campanian examples are from the eastern seaboard of the Atlantic Ocean, showing, in combination with earlier examples from the UK and continental Europe, that *Osedax* had colonized both sides of the northern Atlantic by the Campanian [10]. FMNH PM 459, USNM PAL 768907−9 and USNM PAL 768911 provide the first described instances of *Osspecus* in fossil whales from the eastern Atlantic Ocean. These specimens show that this geographic region was likely continually inhabited by multiple species of *Osedax* from the Eocene to the present day, and this continual habitation potentially extends back to the Campanian (figure 3).

## 5. Conclusion

We present 11 fossil cetacean specimens with newly documented instances of *Osspecus,* the trace boring made by the bone-eating worm *Osedax*. One occurrence from the late Eocene represents the earliest example of *Osspecus* in a fossil cetacean. We also provide the first instances of *Osspecus* in whales from the eastern Atlantic Ocean. Six ichnospecies of *Osspecus* are identified in these cetacean bones, one of which is an ichnospecies newly named here. These examples expand our knowledge of the biogeography and diversity of *Osedax* or *Osedax*-like worms in the Cenozoic and help to show the spatiotemporal continuity of several ichnospecies that are found in both the Cretaceous and the present day or recent fossil record.

Additional studies of modern whalebone are needed to establish the diversity of ichnospecies that are present in today’s oceans and correlate biological species to these ichnospecies in order to understand the relationships of trace fossils to modern phylogenies. A thorough classification of all the available examples of *Osspecus* in the literature into new or known ichnospecies could then provide a more detailed timeline for diversification and distribution of *Osedax* based on established taxonomic and ichnotaxonomic relationships.

Finding earlier Paleogene examples of *Osspecus* in other marine vertebrates would be particularly informative regarding the evolution of the diversity of this clade and how it was affected by the end-Cretaceous mass extinction. Earlier Cenozoic examples would also help to determine whether the habitation of both sides of the Atlantic Ocean basin was continuous from the Late Cretaceous to the present day, or if this continuity of habitation was interrupted by the K-Pg asteroid impact. Fish, as the most prevalent and diverse vertebrate group in all depositional environments across the K-Pg, are likely good candidates for seeking these additional examples and continuing to fill in the temporal and spatial gaps in the evolutionary history of *Osedax*, as are other vertebrate clades that survived the end-Cretaceous extinction, such as turtles, crocodyliforms and terrestrial vertebrates found in marine strata.

## Data Availability

CT scans, scan parameters, and an image of the location of the new ichnospecies holotype in the associated scan are available on MorphoSource [40].
